# Fostering EFL/ESL Students' State Motivation: The Role of Teacher-Student Rapport

**DOI:** 10.3389/fpsyg.2021.754797

**Published:** 2021-09-13

**Authors:** Yanzhi Meng

**Affiliations:** School of Foreign Languages, Xinxiang Medical University, Xinxiang, China

**Keywords:** EFL/ESL students, students' state motivation, teacher-student rapport, self-determination, rhetorical goal theory

## Abstract

It is maintained that one of the significant determining issues of success is motivation, and enhancing EFL/ESL students' motivation is dominant in cultivating their learning in the classroom. Moreover, teachers are reflected as the most noteworthy figure of any scholastic organization and the positive rapport between students and teachers is significant for learners' state motivation. In line with the investigations of teacher-student rapport, principles from positive psychology (PP), and motivational theories such as self-determination and rhetorical/relational goal theory, the present theoretical review seeks this type of relationship and its effects on learners' motivation. Student-teacher rapport results in progressive practices for learners, as well as superior classroom involvement, and motivation. Subsequently, the helpfulness of findings for teachers, learners, materials developers, and teacher trainers are conferred.

## Introduction

Assuming a significant part in learners' education and academic achievement, as shown by research, motivation tends to become weaker and weaker as learners become older and transfer to higher grades and education. A particularly significant stage is the change from primary to secondary education (Murphy and Alexander, 2000; Yeung et al., 2011). In order to capture the role played in the social climate of the class, it is crucial to comprehend the distinctions and longitudinal changes in learners' motivation (Opdenakker et al., 2012). As stated by Corpus et al. (2009), learners' commitment and motivation can be positively influenced by educators who showed inspiring performance. Teachers are likewise key figures, who, especially through class contacts, shape learners' education (Pianta et al., 2012). For instance, researchers have examined learners' motivation as both a state- and a trait-like factor. As described by Christophel (1990), state motivation is a situational concept that alludes to the work put in the direction of a specific assignment domain at a specific point in time. As asserted by (as cited in Goldman et al., 2017), trait motivation is a somewhat steady paradigm that alludes to the general drive of learners toward learning and education. In light of its solid relationship with compelling teaching practices like non-verbal promptness, lucidity, affinity seeking, affirmation, and humor, state motivation is frequently favored by many researchers (Comadena et al., 2007; Goodboy and Myers, 2008; Kerssen-Griep and Witt, 2012).

Language learning is an activity that mainly happens in classes; notwithstanding, few know that motivation develops in these conditions or the ordinary associations between learners and teachers (Ushioda, 2013). There is a need for a study that carries out narrower experimental attention, and which can reveal insight into motivation as it arises in specific exercises and arranged connections since L2 motivation research has generally emphasized learning procedures at an overall level (Ushioda, 2016). The class climate adds to the improvement of learners' motivation and commitment to learning, and further develops learners' scholarly execution (Velayutham and Aldridge, 2013). As a component of the class climate, the teacher-student relationship has been confirmed to be of imperative significance for learners' education (Hughes et al., 2012). Indeed, learners and teachers who arrange a learning relationship in a learning environment to deliver desired learning results are at the focal stage of all instructive frameworks (Pishghadam et al., 2019; Xie and Derakhshan, 2021).

Besides, the acknowledgment that a psychological portrayal of a relationship with an educator can establish a wellspring of motivation is significant since language learning happens at the micro-level of societal action, and includes interactive connections with others inside repeating contexts of use (Henry and Thorsen, 2018). The study of motivation in L2 education goes back to several years ago as some scholars (e.g., Bravo et al., 2017; Busari, 2018; Djaya et al., 2018; Wijnen et al., 2018) pinpointed that motivation is viewed as one of the main persuasive and prominent issues in a person's accomplishment for learning both second or foreign language.

Furthermore, positive teacher-student connections fill in as an outside wellspring of motivational change, thereby adding to dynamic learning practices (Ma et al., 2017). One's motivation and accomplishment in a course might be vigorously impacted by the educator's performance and the students' cooperative activities, as some may have encountered (Passini et al., 2015; Wei et al., 2015). According to social motivation theorists, learners with social help from educators will develop solid motivational convictions that will advance dynamic learning commitment and great execution (Furrer and Skinner, 2003). Indeed, a steady teacher-student rapport can even halfway counter the regularly noticed decrease in learners' self-governing motivation as time goes by (Lapointe et al., 2005). The presumption that the nature of instructor-learner rapport assumes a focal part in advancing motivation and expanding learning returns to speculations from interpersonal psychology, which sees an individual's conduct with regards to conditional causality and reciprocal impacts (Strack and Horowitz, 2012).

Studies on the significance of high-quality teacher-student connections have gotten expanding consideration in 20 years (Roorda et al., 2011). High-quality teacher-student connections give a steady establishment to long-haul learners' education (Hamre and Pianta, 2001). Learners will perform better scholastically and experience more prominent school commitment at the point when they feel their teachers like them (Wang and Eccles, 2013). Numerous inquiries have discovered that learners with close educator rapport are bound to encounter scholastic interest, commitment, accomplishment, self-efficacy, and motivation as opposed to learners with more far-off relations (Sakiz et al., 2012; Tosto et al., 2016).

Learners are bound to miss their classes when they do not feel upheld by their educators (Demir et al., 2019). They are motivated to go to their classes at the point when they have more noteworthy degrees of teacher-learner rapport or trust their educators at higher levels. Learners have more opportunities to develop rapport with their educators when they go to class consistently, and this might advance ceaseless participation. Positive teacher-student connections help learners' transferring to school (Cheung, 2019). When learners were involved in school and had confidence in their capacities, they acclimated it better. Furthermore, learners had more significant levels of motivation when they cooperated with their educators all the more regularly (Liu and Chiang, 2019).

The relations between educators and learners influence learning conditions as many pieces of research have certainly focused on educator cooperation with learners as a recognizable factor to start critical base and motivation for the instructing and learning cycle, to rouse learners' motivation to take a functioning part in the learning climate, to show the teachers' accommodation, and to build a positive class climate that will help learners' education (Bouras and Keskes, 2014).

In light of the fact that learners and teachers are similarly accountable for the fruitful realization of the educational and learning cycles, the rapport between the two is significant (Delos Reyes and Torio, 2020). Thus, they should cooperate to assemble positive learning conditions. By utilizing social practices that are related to learners' positive encounters, educators trigger the foundation of such conditions (Bolkan et al., 2016). Since learning incorporates social, mental, and emotional connections, it could be said that learning includes something beyond simple openness to data. Along these lines, successful education is normally realized inside a positive teacher-learner relationship setting (Strachan, 2020). In spite of the fact that teacher-learner rapport is an indispensable part of any learning situation, the way toward making and keeping a positive rapport is a challenging task for some accomplished educators (Strachan, 2020). Hence, it is extremely fundamental to apprehend the cycles of fundamental successful teacher-learner connections.

Besides, language learning is an intrinsically friendly interaction, considerably more than other scholarly subjects (Mercer and Gkonou, 2020), so the nature of the teacher-learner connection is very significant in the L2 setting (Wang and Derakhshan, 2021). Generally, the language information is educated and utilized adequately through various methods of correspondence (Frymier et al., 2019). Accordingly, there is a great necessity for connections with individual conversationalists (i.e., the educator or friends). Depending on how well educators and learners interact with one another, their instructing and learning encounters can either improve or get ruined, respectively.

Rapport further develops various class domains; explicitly, it advances motivation, criticism, learners' education, correspondence, and obviously the educator's well-being. Learners, who often communicate with a teacher, are more fulfilled and are less inclined to drop out (Wasley, 2006). It is at that point that educators should place more accentuation in building positive rapport in the English as a Foreign Language class as it will certainly be a pivot (Dyrenforth, 2014).

Learners' motivation is a key to numerous compelling teaching practices and their definitive impact (positive or negative) on learners' education results (Frymier, 2016). Two issues have obstructed researchers in testing and appreciating motivational clarifications of educators' correspondence manners. The functional cross-over between the proportions of motivation and emotional learning is the first issue (Goldman et al., 2017). The second issue is the oversimplified perspective on motivation as a paradigm changing just in amount and not in value (Goldman et al., 2017).

The self-determination theory (Deci and Ryan, 2012) and rhetorical/relational goal theory are powerful clarifications of motivation theories that resolve this issue by offering a complete hypothetical structure for understanding educators' correspondence manners (Frymier, 2016). Correspondence analysts must start to accept the self-determination theory to see how educators meet learners' fundamental requirements and how the satisfaction of these necessities works with learners' practices and, eventually, teaching (Bolkan et al., 2016).

Educator promptness, affirmation, and affinity seeking work with positive instructor-learner connections, which, therefore, works with need fulfillment (Frymier, 2016). In particular, when learners feel associated with the instructor by the expansion of these conducts, they are bound to have their relatedness needs fulfilled. Additionally, rhetorical conducts like instructor lucidity and pertinence would almost certainly establish learning conditions that serve to fulfill the necessities of skill and self-sufficiency, opening the entryway for learners to foster their inherent motivation for education.

Upon reviewing the collected works on correspondence and motivation, Frymier (2016) contended that the motivation hypothesis, particularly SDT, gave the best clarification to the connection between instructors' correspondence conduct and learners' education. She contended that compelling educators' correspondence conducts like instantaneousness, lucidity, affirmation, and affinity seeking are likely to be associated with fulfilling learners' requirements for relatedness, skill, and independence, thereby, in a roundabout way, impacts intrinsic motivation.

As stated by Wentzel et al. (2010), a few pieces of research find that learners who foster positive associations with educators are emphatically propelled to seek scholarly greatness. By approvals corresponding to the learner out in the open, to such an extent that the learner who is publicly praised frequently becomes and remains profoundly intrigued by coursework and performs better, educators help to motivate learners' education in the class (as cited in Liu and Chiang, 2019). While literature corresponds that learner-educator rapport is fundamentally identified with learners' motivations, the direction of this connection relies upon the specific communication style (Liu and Chiang, 2019).

Positive teacher-student rapport is strong expediters of a widespread sort of appropriate learner-related results such as engagement, accomplishment, motivation, and confidence (Davis, 2003; Wendt and Courduff, 2018; Derakhshan et al., 2019; Havik and Westergård, 2020; Derakhshan, 2021; Pishghadam et al., 2021). Mainly, a high-quality teacher-student rapport heightens learner motivation; on the contrary, a poor relationship between them is echoed as a failure on students' motivation in the procedure of learning (Wubbels and Brekelmans, 2005; Opdenakker et al., 2012). Outstandingly, longitudinal investigation demonstrated that students who face a route of connectedness with an educator similarly preserve motivation (Hamre and Pianta, 2005). In addition, Joe et al. (2017) stated that teachers' educational provision of learners, and their encouragement of reciprocal self-esteem in the classroom, influence learners' motivation to a great extent.

Regarding EFL/ESL students' state motivation investigation, the impacts of constructive teacher-student rapport have not been studied earlier, and accordingly, it becomes a pertinent topic for this review. Moreover, the main lacuna in this theoretical review based on the researcher's knowledge is that although teacher-learners rapport has been much under investigation in general learning, they are not taken into account from the EFL/ESL perspective (Hagenauer and Volet, 2014). As a result, thanks to the integral relational aspect of language teaching (Mercer and Dörnyei, 2020) and grounded on the current development and flourishing of PP in SLA emphasizing that constructive emotions (Wang et al., 2021), between teacher and learners, must be taken into consideration in L2 teaching and learning and clarifying how and why teacher-student rapport is related to learner motivation has been an enduring trial.

## Theories of Motivation

The self-determination theory and rhetorical/relational goal theory are two of the most generally utilized theoretical methods clarifying the significance of high-quality instructor-learner connections, as a segment of the interpersonal psychology area.

### Self-Determination Theory

The self-determination theory (Deci and Ryan, 2008) clarifies the connection between educator-learner connections and great school revision or scholarly motivation through the satisfaction of three essential mental requirements: the requirement relatedness, the requirement for skill, and the requirement for self-governance. Learners' education and motivation for accomplishment, which is connected to learning, will be expanded if an instructor meets these three fundamental requirements of learners' by showing responsibility, guaranteeing clear structures, and reinforcing the self-governance of learners (Roorda et al., 2011). Self-determination theorists utilize the attachment theory to characterize “commitment” as an emotional part of educator-learner connections. Accordingly, the essential requirement for attachment is firmly connected to the idea of wellbeing (Roorda et al., 2011). The nature of educator-learner connections is thus estimated by the satisfaction of the previously mentioned three essential requirements, which fortify one another and accommodate ideal and healthy development (Bakadorova and Raufelder, 2018).

In particular, SDT envisages that intrinsic motivation relies upon three fundamental mental requirements: the requirement for autonomy, the requirement for competence, and the requirement for relatedness (Deci and Ryan, 2008). Being the apparent wellspring of one's own behavior is known as autonomy which is positively correlated with teacher success and consequently learners' motivation (Derakhshan et al., 2020). People feel autonomous when they disguise their conduct as a declaration of their very own free will (Ryan and Deci, 2002). Feeling compelling in one's continuous collaborations in a social climate is known as competence. People experience competence whenever they experience testing opportunities that permit them to relay their actual capabilities (Deci and Ryan, 2008). Perceiving an association with others is known as relatedness. Individuals need to have secure associations with others for relatedness to be fulfilled. These significant interpersonal associations might be developed with the educator and/or different learners in the learning setting. Thus, relatedness assumes a fundamental part in encouraging commitment, particularly when initial intrinsic, or inborn, motivation is deficient (Ryan and Deci, 2009). Relatedness is experienced when people foster a feeling of belongingness with their companions, or with others for whom they have high regard (e.g., Moller et al., 2010; Beachboard et al., 2011).

All the more explicitly, when learners experience relatedness with instructors, they get more prominent joy from learning exercises in a school subject and are bound to see themselves as more skilled in this subject (Deci and Ryan, 2000). Participating in communications and keeping up with connections is subject to interpersonal motives, from a socio-motivational viewpoint. Certainly, individuals can be variously motivated to connect. The two fundamental elements of interpersonal motives are agency and communion. The former identifies with attention on self-interest, accomplishment, and individual impact, and the latter identifies with an emphasis on enthusiasm and social cooperation (Horowitz et al., 2006).

### The Rhetorical/Relational Goal Theory

Proposed by (as cited in Zheng, 2021) to perceive how the cycle of instructional correspondence works, the rhetorical/relational goal theory is a hypothesis in the domain of instructional correspondence. In light of this theory, teachers and learners in the class have rhetorical and relational objectives that they wish to accomplish. The significant obligation is the educators' as they are relied upon to oversee both relational and rhetorical necessities simultaneously through their manner decisions to satisfy the class's needs; thus, ideal learning can occur when these objectives are attained and learners' needs are fulfilled.

It is important to mention that the rhetorical and relational practices of educators fill various roles. For example, teachers utilize rhetorical educational correspondence practices to advance compelling educating and influence convictions, perspectives, and practices of students in the class through molding their planned instructional messages (Beebe and Mottet, 2009). Then again, as stated by Myers (2008), educators utilize relational instructional correspondence practices to trigger the development of a commonly formed proficient relationship and rapport with their learners. Certainly, to achieve good results in any learning setting, educators should utilize a combination of rhetorical and relational practices (Myers et al., 2018).

## Teacher–Student Rapport

An amicable educator-learner relationship identified with delight, association, regard, and common trust is known as rapport (Delos Reyes and Torio, 2020). Being significantly relationship-based, it is a relational bond during the education cycle (Frisby and Housley Gaffney, 2015). As stated by Frisby et al. (2016), rapport, in contrast to other educational correspondence factors, is less examined. However, since rapport is an inevitable part of instruction as well as the fact that learners' learning starts from it, it is perhaps the most important component of educational correspondence. Rapport can be built by educators in the class by advancing free expression, respecting learners' mentalities, giving fitting criticism, utilizing humor, displaying eagerness in learners' education, and being delicate and enthusiastic (Weimer, 2010).

## Implications and Future Directions

The current review of literature has some implications for instructive situations. Consequently, it can be of importance to several participants in the educational setting, together with teachers, teacher trainers, materials developers, and those who are responsible for employing teachers, and those preparing teacher professional development programs.

The current review can be instructive and beneficial for language educators as they ought to attend workshops that are intended to emphasize the relational part of the class climate to learn explicit techniques which will work with positive associations with learners (Terry, 2006). Given the significance of social practices and the impression of the connections on learners' education, educator training in building connections to accomplish positive results is probably an advantageous endeavor.

Based on this theoretical review, it is confirmed that teacher-student rapport not only boosted the rudimentary teaching development but also supported the growth of students' motivation on the way to success. It is the responsibility of the teachers to build students' motivation and inspire them to create positive approaches toward learning and their behavior is thoroughly correlated with student motivation and presentation and teacher support through interpersonal relationships is interconnected to students' subject-related motivational aspects (Yildirim, 2012). The more, the students face teacher support, the greater their degree of motivation in the route of language education which is concerning their social-emotional regulation (McElhaney et al., 2008).

From an informative perspective, the present review sheds light on the importance of student-teacher rapport as it inspires and motivates the educator to articulate the new teaching methods and review their ideas to construct a positive relationship with students to boost motivation that results in their engagement and achievement. It is worth noting that teachers who have a constructive relationship with their learners generate a classroom atmosphere that improves learning and runs into learners' emotional and scholastic desires. Positive student-teacher rapport provides the groundwork for efficacious alteration to the educational situation for learners at the inception of their formal teaching. Learners, who reflect that their educator does not support them in the classroom, have low attentiveness and they are less motivated and active in the learning setting (Tyler and Boelter, 2008). In the same way, when educators collaborate with learners and have a respectful and sympathetic attitude toward learners, their stress diminishes and they can fully focus on their lessons with the high motivation that is congruent with Mercer and Dörnyei (2020), who evinced that the L2 Motivational Self System creates a brilliant route of attentiveness and engagement. Moreover, teachers should develop their interpersonal behavior through cultivating their proximity, i.e., their physical and emotional confidence to learners and such performances will generate a constructive reaction from learners toward their educators, firmly functioning to surge learners' pleasure by articulating confidence, and being supportive (Beebe and Mottet, 2009). Accordingly, by adopting interactional manners, and cooperating with learners not only in the class but also before and after the class, educators are supposed to accomplish higher motivation leading to educational and emotional upshots.

As for students, this line of review can be of importance as it increases their consciousness and awareness of the fact that an educational method is a co-built event whose attainment is not wholly the responsibility of educators. Indeed, learners are correspondingly dominant in providing an abundant way for their achievement to happen. They try to begin a sociable rapport with their teachers, and this closeness enhances their confidence and motivation. When learners are acquainted with their dynamic role in the learning process, they show more motivation and commitment in their performances.

The next participants who can use this review are teacher trainers who can nurture the knowledge of novice educators regarding teacher-student rapport as they can propose workshops, conferences, and other preparation developments in which educational, emotional, and interpersonal features of education are correspondingly taught. For better upshots, governments need to fill the lacuna between government and language school organizations. Appropriate supervision and psychoanalysis should be provided for students at diverse levels of their learning development to motivate them in language learning. More in detail, teacher mentors can provide a chance for educators to discern the implication and practice of teacher-student rapport in the achievement of their careers. Additionally, materials developers can take advantage of this type of research in a way to design courses that consider the teacher-student rapport as a core component of education that emphasizes rapport in the process of accomplishing tasks and in such a way that educators can focus on their clearness, rapport with students, and authority.

In conclusion, portfolios, philosophical papers, and think-aloud procedures can be employed as well to perceive the mental procedures that teachers and learners come across. Likewise, the interpersonal rapport of teachers and learners should be studied from the points of view of both teachers and learners. Further research can be utilized on the opinions of administrators, teacher trainers, too. In addition, enthusiastic scholars are recommended to scrutinize the influence of educators' experience level, academic experiences, age, and gender in this domain.

## Author Contributions

The author confirms being the sole contributor of this work and has approved it for publication.

## Conflict of Interest

The author declares that the research was conducted in the absence of any commercial or financial relationships that could be construed as a potential conflict of interest.

## Publisher's Note

All claims expressed in this article are solely those of the authors and do not necessarily represent those of their affiliated organizations, or those of the publisher, the editors and the reviewers. Any product that may be evaluated in this article, or claim that may be made by its manufacturer, is not guaranteed or endorsed by the publisher.
